# Simplified programming and control of automated radiosynthesizers through unit operations

**DOI:** 10.1186/2191-219X-3-53

**Published:** 2013-07-15

**Authors:** Shane B Claggett, Kevin M Quinn, Mark Lazari, Melissa D Moore, R Michael van Dam

**Affiliations:** 1 Crump Institute for Molecular Imaging and Department of Molecular and Medical Pharmacology David Geffen School of Medicine, University of California Los Angeles, Building 114, 570 Westwood Plaza, Los Angeles 90095, CA, USA; 2 Sofie Biosciences, Inc., 6162 Bristol Parkway, Culver City 90230, CA, USA

**Keywords:** Radiochemistry software, Synthesis automation, Positron emission tomography, Unit operations

## Abstract

**Background:**

Many automated radiosynthesizers for producing positron emission tomography (PET) probes provide a means for the operator to create custom synthesis programs. The programming interfaces are typically designed with the engineer rather than the radiochemist in mind, requiring lengthy programs to be created from sequences of low-level, non-intuitive hardware operations. In some cases, the user is even responsible for adding steps to update the graphical representation of the system. In light of these unnecessarily complex approaches, we have created software to perform radiochemistry on the ELIXYS radiosynthesizer with the goal of being intuitive and easy to use.

**Methods:**

Radiochemists were consulted, and a wide range of radiosyntheses were analyzed to determine a comprehensive set of basic chemistry unit operations. Based around these operations, we created a software control system with a client–server architecture. In an attempt to maximize flexibility, the client software was designed to run on a variety of portable multi-touch devices. The software was used to create programs for the synthesis of several ^18^F-labeled probes on the ELIXYS radiosynthesizer, with [^18^F]FDG detailed here. To gauge the user-friendliness of the software, program lengths were compared to those from other systems. A small sample group with no prior radiosynthesizer experience was tasked with creating and running a simple protocol.

**Results:**

The software was successfully used to synthesize several ^18^F-labeled PET probes, including [^18^F]FDG, with synthesis times and yields comparable to literature reports. The resulting programs were significantly shorter and easier to debug than programs from other systems. The sample group of naive users created and ran a simple protocol within a couple of hours, revealing a very short learning curve. The client–server architecture provided reliability, enabling continuity of the synthesis run even if the computer running the client software failed. The architecture enabled a single user to control the hardware while others observed the run in progress or created programs for other probes.

**Conclusions:**

We developed a novel unit operation-based software interface to control automated radiosynthesizers that reduced the program length and complexity and also exhibited a short learning curve. The client–server architecture provided robustness and flexibility.

## Background

Positron emission tomography (PET) has become a key tool in medical imaging with primary applications being cancer diagnosis, staging, and monitoring of treatment [1-3], with additional applications in many other disciplines including neurology [4,5], cardiology [6,7], and pharmacology [8,9]. As the number of applications and PET probes has grown, so has the need to produce an ever-increasing variety of radiolabeled compounds for both preclinical and clinical uses. Probes are traditionally synthesized by skilled radiochemists using specialized equipment and facilities that reduce their radiation exposure when working with large quantities of short-lived isotopes necessary to produce a final dose sufficient for imaging a human. In recent years, the development of automated radiosynthesizers that can produce a variety of different probes with minimal human intervention or radiation exposure [10,11] has aimed to simplify routine synthesis of PET probes, especially for the clinic. As such, these synthesizers can be operated by technicians and do not require a highly trained radiochemist. Additionally, some automated systems can be configured to prepare different PET probes and thus also act as valuable tools for researchers developing new synthesis protocols for novel probes. In order to be useful to chemists, these systems must also provide an intuitive and easy-to-use software interface for the creation and modification of synthesis programs.

There are a variety of radiochemical synthesizers on the market with a range of features and capabilities [12]. Examples include IBA's Synthera® [13], GE's FASTlab [14] and TRACERlab [15], Eckert & Ziegler's Modular-Lab [16] and PharmTracer [17], and Siemens' Explora® GNs [18]. However, the software that drives these systems tends to be overly complex and requires a deep understanding of the system internals. For example, synthesis programs on the Synthera® are composed of low-level operations such as switching individual valves within the main system or the disposable cassette (integrated fluidic processor). As a result, the program to perform the relatively simple synthesis of 2-[^18^F]fluoro-2-deoxy-d-glucose ([^18^F]FDG) requires a program that consists of 227 steps (determined from the protocol supplied by the manufacturer at the time of installation). These programs are written in a scripting language that may not be intuitive to a radiochemist or technician without computer programming experience and hence require the investment of significant time and energy to master. The radiochemist is also required to have a detailed understanding of the mechanisms of fluid transfer and the fluidic architecture of the system. The Modular-Lab software enables programs to be built graphically as flow charts, rather than written as scripts, but the flow chart elements consist of low-level hardware operations or steps to activate visual cues that are presented on an engineering schematic of the system during production runs. The complexity of developing synthesis programs is not a concern for routine production, where fixed programs are run on a regular basis, but becomes a significant hurdle for radiochemists, who frequently develop and optimize automated synthesis protocols for novel probes. Several previous works have attempted to reduce the complexity of developing new synthesis protocols by implementing higher-level unit operations or macros but still required the user to be familiar with the low-level system details and write syntheses in cryptic scripting languages [19-21].

We describe here the development of a software package [22,23] to run the ELIXYS (Sofie Biosciences, Culver City, CA, USA), a disposable cassette-based, automated multi-reactor radiosynthesizer [24-28] that is designed for both the development of new synthesis protocols and routine probe production. To facilitate the creation and modification of programs, this software is based on high-level unit operations designed to make intuitive sense to a chemist. A small number of adjustable parameters for each of these operations provide considerable flexibility to implement diverse syntheses and optimize conditions but do not require a detailed understanding of fluidics and low-level hardware architecture. Examples of these unit operations are ‘ADD’ which adds any reagent to any reaction vessel, ‘REACT’ which performs a reaction under sealed conditions, and ‘TRANSFER’ which transfers the contents of one reaction vessel to another with an optional cartridge purification step. The client software uses a drag-and-drop interface to further simplify the programming process and is designed to run on multi-touch tablets and phones. The server software that runs the instrument supports multiple client connections simultaneously, to allow others to watch the current synthesis run for increased transparency and oversight, and is tolerant of failures of client devices without impacting a production run in progress. We describe the software architecture and user interface design to illustrate these differences, evaluate the software in terms of its ease of use, and demonstrate the successful synthesis of several PET probes. It is our hope that this new software will empower radiochemists to focus on chemistry rather than engineering and to develop and produce new probes more quickly.

## Methods

### Hardware system overview

**Figure 1:**
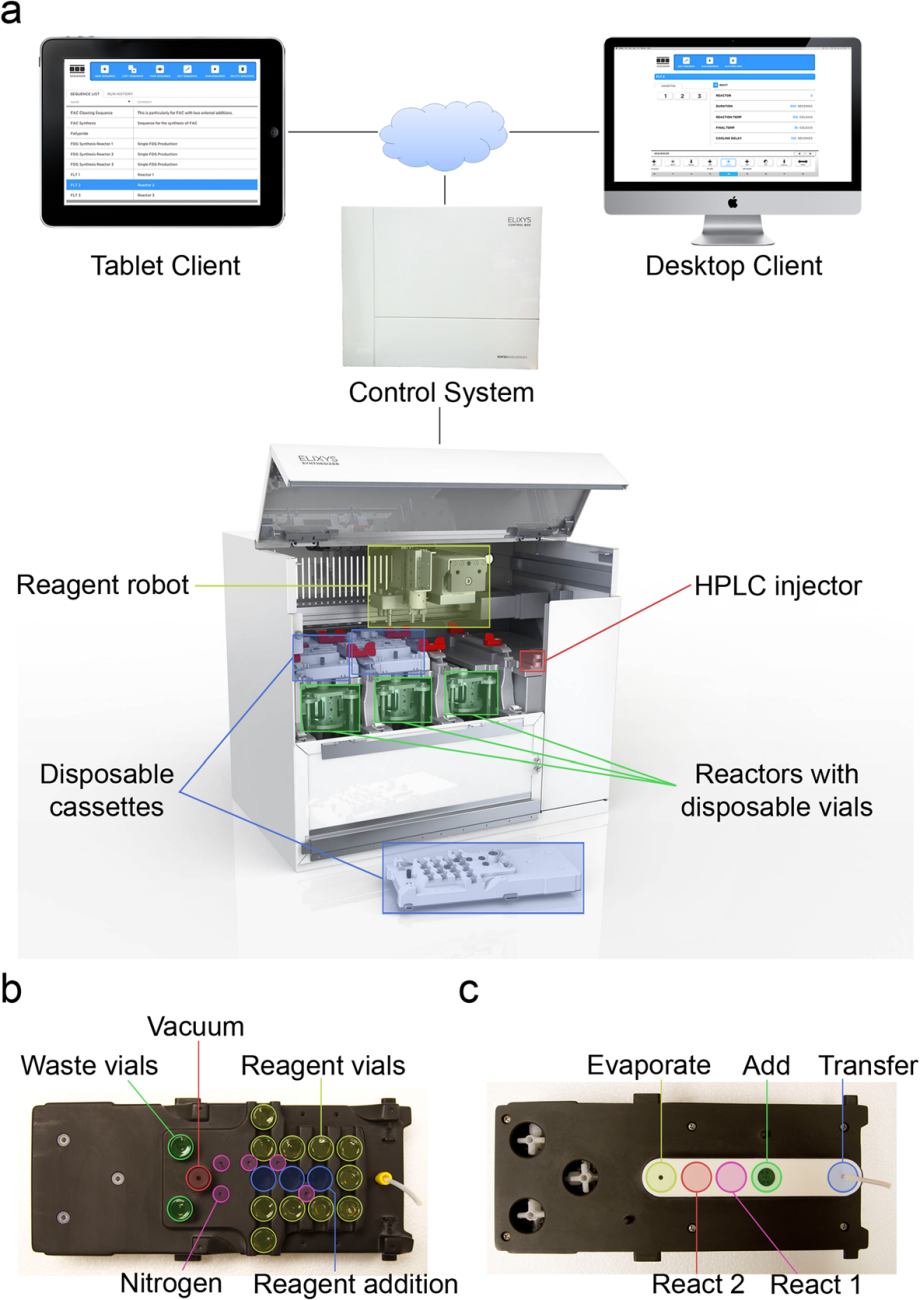
**Overview of the ELIXYS radiosynthesizer. (a)** A variety of client devices communicate with the server (housed in the control system), which in turn drives the hardware in the synthesis module via the controller. **(b)** Top view of disposable cassette showing the location of the eleven reagent vials and the three reagent addition positions. **(c)** Bottom view of the disposable cassette showing the five stations on the gasket where the reaction vessel seals to perform various functions.

The ELIXYS platform (Figure 1) is described in detail in a separate publication [29]. Briefly, it consists of three reaction vessels, each of which can be actively heated and cooled. A camera is provided to monitor the contents of each vessel. Each reaction vessel is moved along two axes using a combination of a servomotor and pneumatics to provide dynamically reconfigurable fluid pathways and allow the vessel to be sealed against several different positions on a gasket that is located on the underside of a disposable cassette. Positions are available for reagent addition (with connections from the previous cassette, reagent vials, or external delivery lines), evaporation (with connections to inert gas and the vacuum system), transfer (with connections to inert gas and a dip tube), and sealed reactions (with no fluidic connections). The motion in the system enables the reaction vessels to safely perform sealed reactions at high temperatures and pressures (e.g., acetonitrile at 180°C) without loss of solvent [25,27]. A total of up to 33 sealed reagent vials with liquid capacities of up to 3 mL can be stored upside down in the cassettes and added on demand to any of the three reaction vessels via a Cartesian robot located on top of the instrument and equipped with a vial gripper. During delivery, the gripper moves the vial to addition positions where needles provide a fluid path to the delivery position on the underside of the cassette. The three disposable cassettes contain all wetted fluid paths used during the synthesis to eliminate the need for cleaning and allow for rapid turnover of the system between syntheses. Cartridges can be plumbed between reaction vessels for intermediate purification or for final preparation of probes that do not require high-performance liquid chromatography (HPLC) purification. A small number of disposable stopcock valves in each cassette control flow through the cartridges for trap and elute operations. An HPLC injection valve and loop are also integrated into the instrument to facilitate interfacing with an external semi-preparative HPLC purification system. Three solid-state radiation sensors are mounted to allow relative estimations of the amount of activity in each reactor.

### Software overview

**Figure 2:**
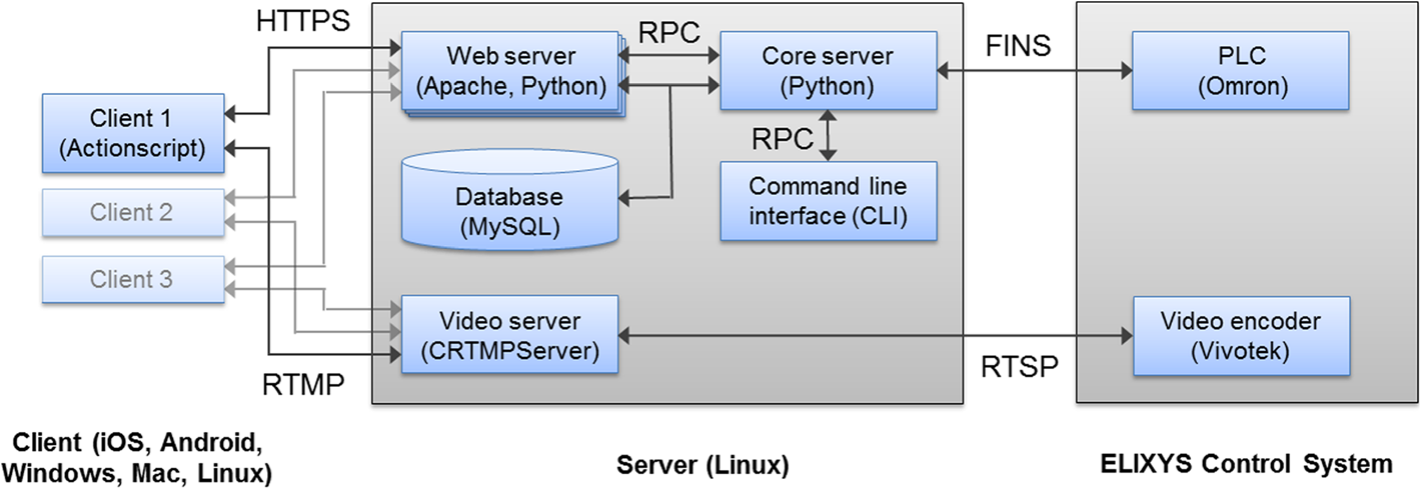
Overview of the ELIXYS software architecture.

The software was designed as a collection of individual applications (Figure 2) that have been divided along functional lines to increase the reliability of the system and reduce the likelihood that a failure in one application will negatively affect the outcome of a production run. The system software can be divided into three tiers: one or more client devices communicate with a server which in turn relays information to and from the controller that drives the low-level operations of the system hardware. Since the failure of a synthesis run can have far-reaching negative effects on both preclinical research scheduling and willingness of patients and doctors to participate in clinical trials, attention was paid to the robustness and fault tolerance of the software stack to reduce the likelihood of it being the source of run failure.

The ELIXYS client is the application the radiochemist uses to operate the system and enables creating or editing a synthesis program, running a program, as well as observing a run that is currently in progress. Flash ActionScript (Adobe Systems, San Jose, CA, USA) was chosen as the programming language for the client in order to provide computer platform independence and maximize the diversity of possible devices that can be used to control the system. Although primarily designed for use on multi-touch tablets such as Apple's iPad and iPhone and devices running Google's Android operating system, the client software can also run on Windows, Mac, and Linux desktops. The client must be installed on the iPad and iPhone as an application; all other platforms provide the option of either installing as an application or running in the web browser. Standard networking protocols have been chosen to maximize the ability of the client–server communication to pass through firewalls. Secure hypertext transfer protocol (HTTPS) is used to transmit all information with the exception of video which is sent using Adobe's real-time messaging protocol (RTMP).

The second tier, i.e., the ELIXYS server, is responsible for the actual execution of the synthesis program and has been designed and built with maximum reliability in mind. To this end, open-source packages that have been used and tested by the community for years were chosen as the basis for the server. Additionally, all information about the state of each client application is stored on the server, so nothing will be lost even if a critical failure (e.g., battery loss and software crash) occurs with the tablet or phone. The server is driven by the client device but acts independently once the production run has started to make the system resilient to intermittent network connectivity or failures of the client device. The ELIXYS server is composed of five main applications:

1. *Web server*. The Apache HTTP server (Forest Hill, MD, USA) is responsible for all client communication except video and was chosen because it is one of the most widely used web servers available. A module written in Python (Wilmington, DE, USA) handles viewing and editing programs and only communicates with the core server for operations related to production runs.

2. *Core server*. An application written in Python that is responsible for running a program and communicating with the programmable logic controller (PLC) in the radiosynthesizer that monitors and controls the state of the hardware. The PLC constitutes the third tier of the software. The core server code has been separated from the web server to remove the overhead of program viewing and editing and to insulate it from any failures that might occur while processing client requests. All communications between the web and core servers are accomplished using remote procedure calls.

3. *MySQL server*. All synthesis programs and user information as well as the complete production run history are stored in a MySQL database (Redwood City, CA, USA), a widely used, reliable, open-source relational database.

4. *Video server*. Three live video feeds from the reaction vessel cameras are generated by a hardware encoder within the ELIXYS synthesizer as real-time streaming protocol streams and are converted to the Flash-compatible format RTMP by C++ RTMP Server [30] and published for simultaneous consumption by multiple client devices. Note that for licensing reasons the commercially available ELIXYS system uses the commercial version of C++ RTMP Server named EvoStream (San Diego, CA, USA) [31].

5. *Command line interface*. A terminal-based command line interface provides a way to monitor the status of all hardware components and offers a mechanism to control the system directly at a low level. Although not needed by or intended for end users, this application is useful for software developers and maintenance technicians.

Security is always a concern for client–server systems and becomes even more critical when the server drives a radiochemistry system. The software stack described above has been chosen to minimize the attack surface by selecting components that have a history of being successfully deployed in insecure environments. HTTP communication between the client and server is sent over a channel that is encrypted using the industry standard transport layer security. The full source code is available online in a Github repository [32].

### Programming with unit operations

Radiochemistry systems are typically programmed at the level of individual valves and other components, requiring a detailed understanding of the underlying system hardware. Such an approach necessitates a significant learning curve to become familiar with the particular system details and the programming language/interface such that creation and optimization of a desired synthesis can be accomplished. The software package that we developed introduces a new paradigm that strives to eliminate these unnecessary complexities and instead allows the end user to describe the synthesis in terms that we believe make intuitive sense to a chemist or radiochemist that may have no prior experience with automated systems. A new synthesis protocol is created in two stages: (1) the reagents that will be used in the synthesis are described, and (2) the program is built by stringing together an ordered sequence of unit operations. The user can switch back and forth between these stages with the caveat that the unit operations cannot be fully configured until the relevant reagents have been defined. Rather than creating all new synthesis programs from scratch, it is also possible to copy an existing synthesis protocol and use that as the starting point.

**Figure 3:**
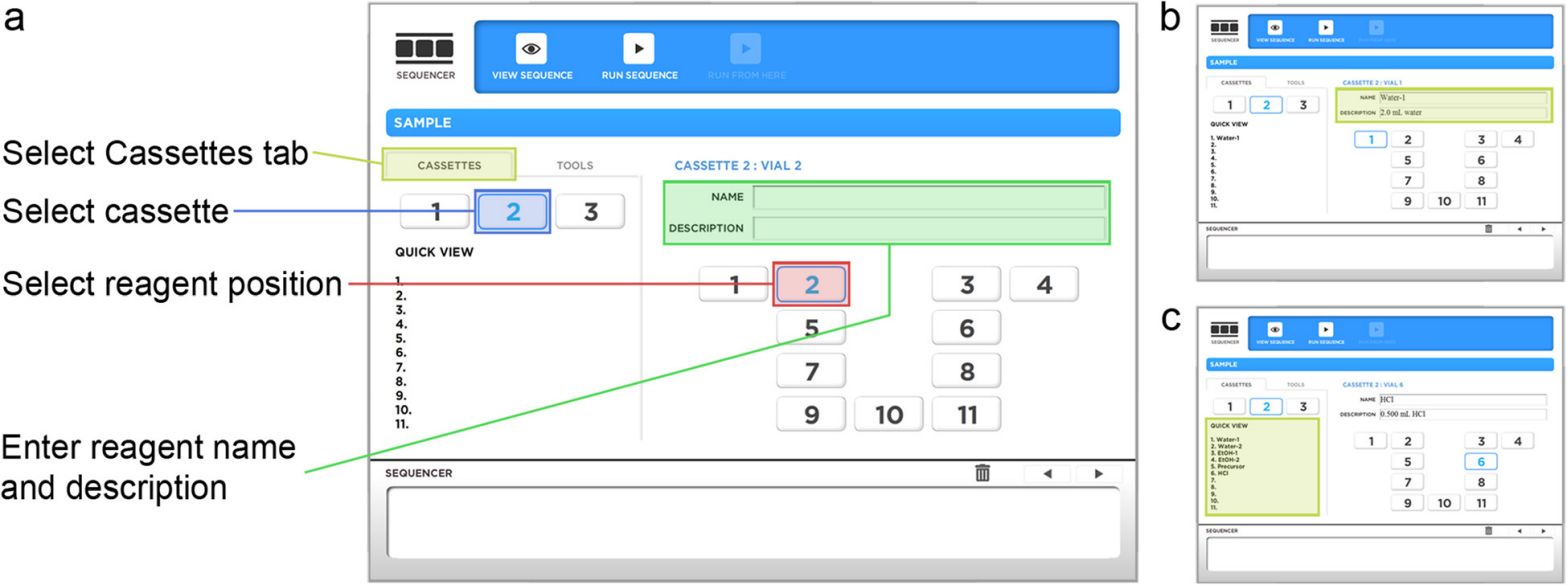
**First step in creating a new synthesis protocol.** The first step in creating a new synthesis protocol is to define the reagents by specifying their storage locations in the three disposable cassettes. **(a)** Key fields in the reagent editing screen. **(b)** Reagent name and description are entered in the two edit boxes above the layout diagram. **(c)** The quick view list displays the names of the reagents in the currently selected cassette at a glance.

Reagents used in a synthesis are mapped to specific vial storage positions in the three disposable cassettes (Figure 3). The user first selects the ‘CASSETTES’ tab and then chooses one of the three cassettes and a reagent position within that cassette. Two edit fields above the cassette diagram allow the user to enter a human-readable name for the reagent and an optional, longer description. The reagent is thereafter referred to by name to alleviate the need to remember the actual position. The description field can be used to make notes about the reagent composition and can include item or lot numbers to simplify reagent tracking and reordering. The quick view list on the CASSETTES tab shows all of the reagents in the selected cassette at a glance. The user will still need to make sure each reagent vial is placed in the proper position at the start of a run, a process that could be simplified in commercial kits by color-coded vials and cassette positions or pre-installing some or all reagents in the cassettes.

**Figure 4:**
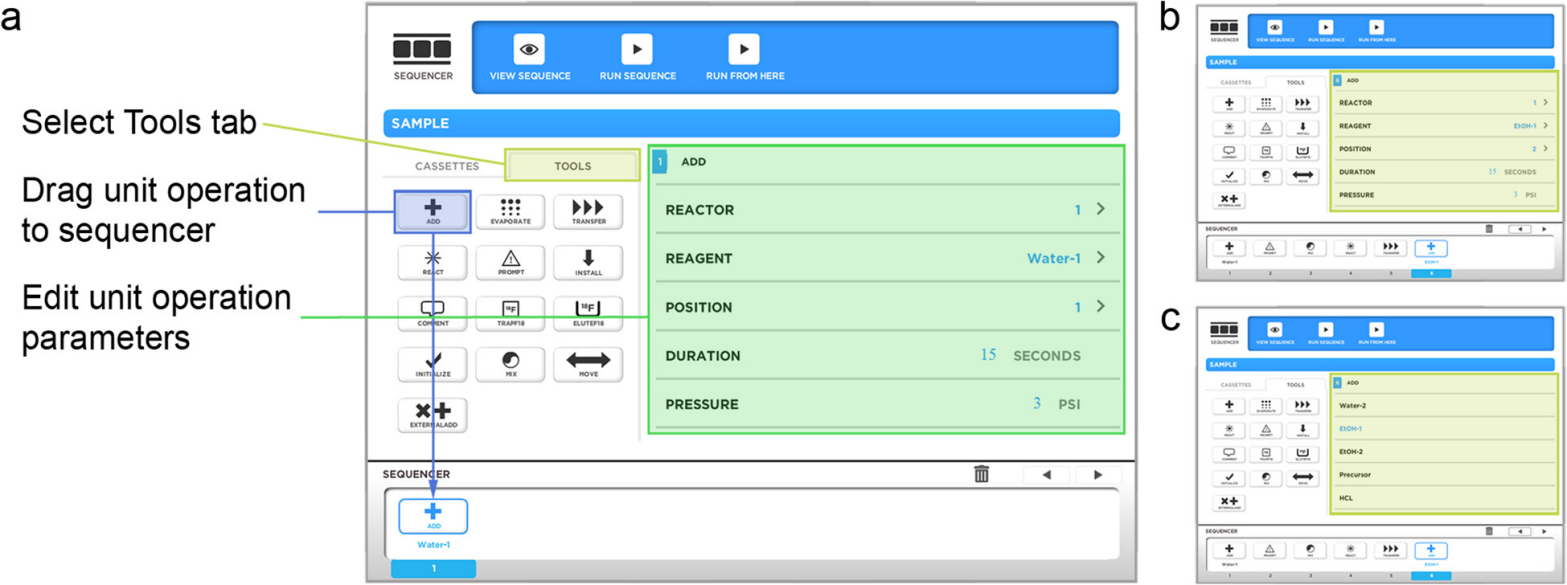
**Second step in creating a new synthesis protocol.** The second step in creating a new synthesis protocol is to define a series of unit operations that describe the synthesis steps and then configure the parameters associated with each individual unit operation. **(a)** Key fields in the program editing screen. **(b)** Each unit operation has a small number of relevant parameters the user can configure. **(c)** A list view allows the user to choose a value for parameters that have a number of possible options (e.g. reactor, reagent).

After the reagents are defined, the synthesis program is created by stringing together a series of unit operations and configuring the parameters associated with each. A complete list of the available operations is shown in Table 1. These include familiar actions, such as adding a reagent to a reaction vessel, evaporating the solvent from a reaction vessel, or transferring the contents from one reaction vessel to the next. Each unit operation has a small number of parameters that configure the behavior of that operation and allow the radiochemist to optimize the synthesis via fine-tuning of heating times and temperatures, stirring, liquid transfer pressures and times, implementation of intermediate purifications, etc. For example, when adding a reagent to a reaction vessel (ADD operation), the user needs to select the source reagent, target reaction vessel, and which of the two possible addition needles to use; two separate needles are available to prevent cross-contamination when adding incompatible reagents. Additional parameters such as the inert gas driving pressure and delivery time are automatically set to default values but can be adjusted, for example, when dealing with viscous reagents that require a little more time to flow from the reagent vial to the reaction vessel. The source reagent is chosen by its name. Unit operations are added to the program by simply dragging them from the ‘TOOLS’ tab and dropping them on the ‘FILMSTRIP’ view of the program (Figure 4). Included in the TOOLS tab are operations that pause the synthesis to allow users to take radioactivity measurements of a reaction vessel using a dose calibrator or liquid samples for analysis to facilitate probe development and synthesis optimization. A unit operation that can automatically measure the relative activity level of each reaction vessel using the integrated radiation sensors is currently under development. The ‘PROMPT’ operation causes a pop-up message to be generated during a production run with instructions for the user when non-automated tasks are required, such as the preparation of a sensitive reagent for addition through an external addition line. The parameters associated with the currently selected unit operation are listed in the main editor window and can be changed using the user interface. The filmstrip view can also be used to rearrange and delete unit operations using the drag-and-drop interface. Parameter values that are not set or that are incorrectly set (e.g., outside the range of permitted values) will generate error messages, and visual indicators will highlight the problem parameter(s) so they can be corrected.

**Table 1:** Unit operations and select parameters

**Unit operation**	**Definition**	**Parameter**	**Description**
ADD	Adds a reagent to a reaction vessel	Reagent	Reagent to add
		Reactor	Reactor to which the reagent will be added
		Delivery position	Choice of two separate delivery positions
EVAPORATE	Evaporates the contents of a reaction vessel	Reactor	Reactor to evaporate
		Evaporation temp	Temperature to heat to during evaporation (°C)
		Evaporation pressure	Inert gas pressure to use when evaporating (psi)
		Final temp	Temperature to cool to after evaporation (°C)
		Duration	Time to evaporate after reaching temp (s)
		Stir speed	Rate of stirring during evaporation
TRANSFER	Transfers the contents of one reactor to the next (optionally through a purification cartridge)	Source reactor	Reactor to transfer from
		Target reactor	Reactor to transfer to
		Mode	‘Trap’ to send to waste or ‘Elute’ to send to the next reactor
REACT	Seals the reactor and heats	Reactor	Reactor to heat
		Reaction temp	Temperature to heat to during reaction (°C)
		Reaction position	Choice of two separate reaction positions to avoid cross-contamination if multiple sealed reactions are performed in the same vessel
		Duration	Time to react after reaching temperature (s)
		Final temp	Temperature to cool to after reaction (°C)
		Stir speed	Rate of stirring during reaction
PROMPT	Pauses the sequence run and prompts the user	Message	Message to display when prompting the user
INSTALL	Moves the reactor to the install position for reaction vessel removal and/or installation and prompts the user	Reactor	Reactor to move to the install position
		Message	Message to display when prompting the user
COMMENT	User comment	Comment	User-specified comment for documentation purposes only (no action performed)
TRAPF18	Traps [^18^F]fluoride on a QMA cartridge	Reactor	Reactor where the QMA cartridge is located
		Cyclotron flag	Specifies if the cyclotron or ELIXYS will push the solution
		Duration	Trap time (s)
		Pressure	Pressure of inert gas to use when trapping (psi)
ELUTEF18	Uses a reagent to elute [^18^F]fluoride off a QMA cartridge	Reactor	Reactor where the QMA cartridge is located
		Reagent	Reagent with which to elute the contents of the QMA cartridge
		Duration	Elute time (s)
		Pressure	Pressure of inert gas to use when eluting (psi)
MIX	Mixes the contents of a reactor by stirring	Reactor	Reactor to mix
		Duration	Mix time (s)
		Stir speed	Rate of stirring while mixing
EXTERNALADD	Allows the user to externally add a reagent via tubing	Reactor	Reactor to externally add the reagent
		Reagent name	Name of the reagent to add
		Message	Message to display when prompting the user
TRANSFERTOHPLC^a^	Transfers the contents of the reactor to the HPLC injection loop	Source reactor	Reactor to transfer from
		Mobile phase	Reagent containing the mobile phase
MEASURERADIATION^a^	Measures the radiation levels	Reactor	Reactor to measure

*QMA* quaternary methylammonium. ^a^These unit operations are currently under development.

A detailed list of the low-level hardware operations that make up each high-level unit operation is given in Additional file 1 along with a full list of configurable parameters associated with each unit operation. The ability to tweak these parameters provides considerable flexibility while the use of default values for many fields reduces the complexity in normal use cases. Unit operations also increase the robustness of the system by guaranteeing that hardware operations are executed in a safe order to prevent damage to the system that can occur if the robots are used incorrectly. In essence, the removal of low-level programming reduces the possibility for user error while focusing probe development on parameters that can have the most profound impact on yield and purity.

### Running a synthesis

**Figure 5:**
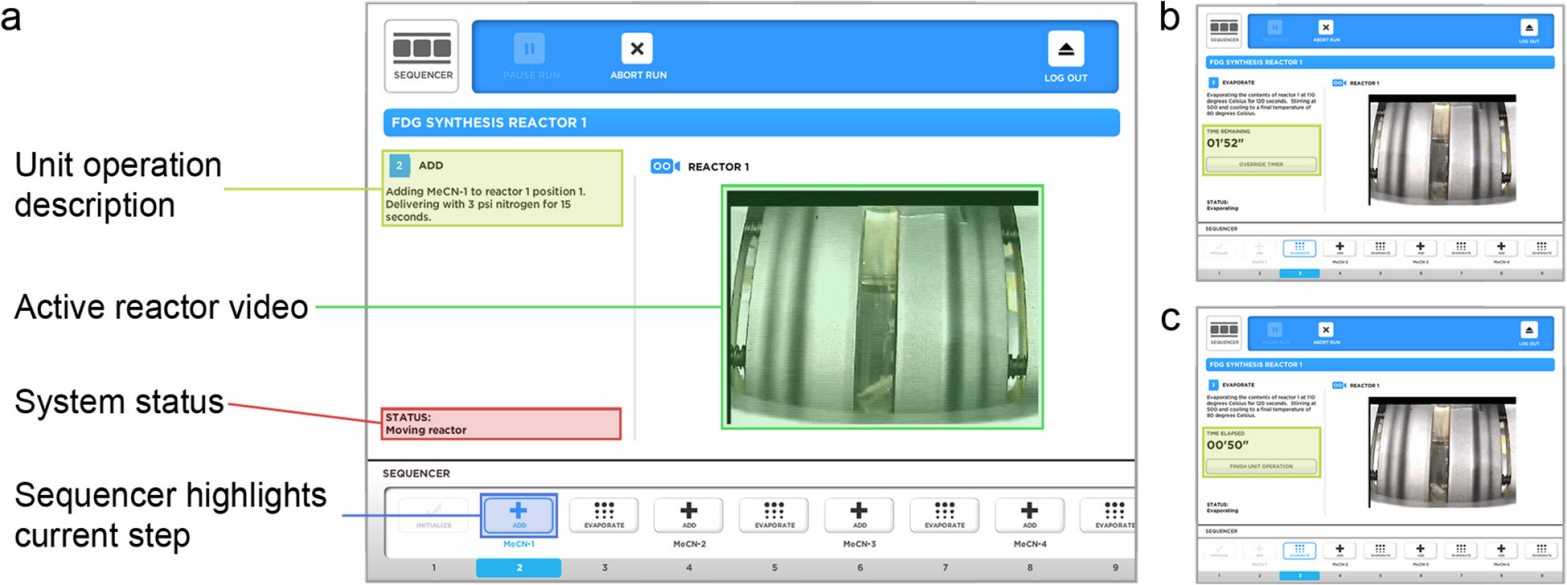
**Information relevant to the current unit operation during a synthesis run.** It is displayed to the user and includes a live video stream from the active reactor(s). **(a)** Key fields in the program running screen. **(b)** An ‘OVERRIDE TIMER’ button allows timed operations to be adjusted dynamically during program development. After pushing this button, the time switches to display the total elapsed time spent performing the current step. **(c)** This button changes to ‘FINISH UNIT OPERATION’ when pushed to allow the radiochemist to indicate step completion once the desired amount of time has elapsed.

The client user interface was designed with the goal of achieving a high level of simplicity and user focus while running a synthesis. To this end, only information that is relevant to the current unit operation and of interest to the end user is shown (Figure 5). This information includes a plain-text description of the current unit operation, the system status, the amount of time remaining for timed steps, and live video from the active reactor(s). Several forms of feedback and control assist with development and optimization without cluttering the user interface. For example, unit operations that contain a time delay specified by the user during program creation (e.g., REACT and EVAPORATE) can be manually overridden during the run to lengthen or shorten the operation as needed. The live video of each reaction vessel can provide valuable feedback to the radiochemist on the state of the synthesis by enabling the contents of the reaction vessel to be continuously monitored in real time.

**Figure 6:**
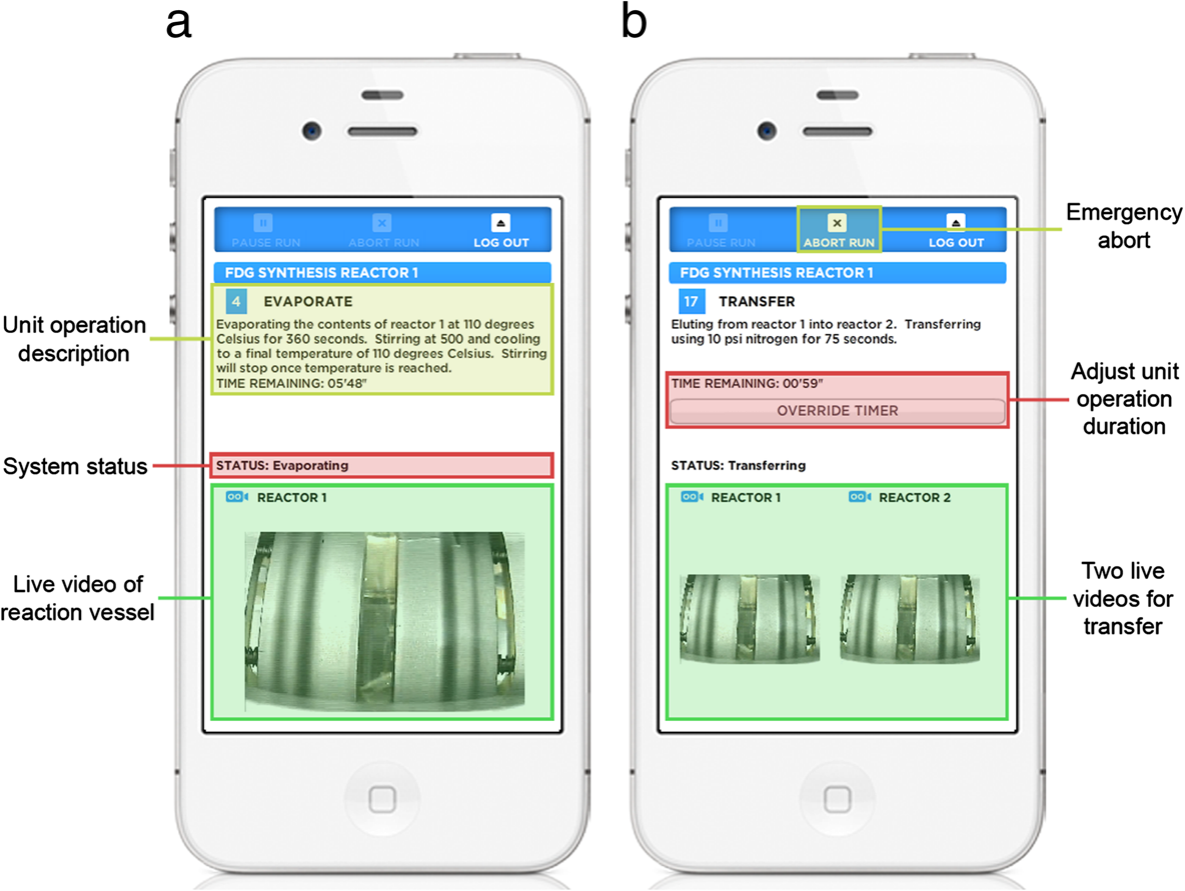
**A small-screen version of the client application.** It allows the user to monitor the run remotely from a smartphone and provides the same control during a run as the tablet version. **(a)** Key fields in the program running screen. **(b)** The small-screen application allows the user to adjust the duration of a step and abort the run.

A small-screen version of the client application has been created for use on smartphones (Figure 6) [23]. This version of the application supports viewing the run that is currently in progress and can provide valuable information regarding the state of the run as long as the radiochemist is within range of his cellular data network. Additionally, the software can be configured with a Twilio account (San Francisco, CA, USA) [33] to send SMS messages to any cellular phone at key points in the synthesis such as run start, completion and failure, and at user-defined points using the COMMENT unit operation.

For purposes of generating a batch record, all details of the system state are captured four times per second and logged to the database along with detailed information about the current synthesis step. We are in the process of developing tools to view the run history and export formatted batch records. In addition, the software includes user access controls and will soon include versioning of synthesis programs to help facilitate current good manufacturing practice (cGMP) compliance.

### [^18^F]FDG synthesis

The reagents and synthesis program for [^18^F]FDG on the ELIXYS system are described in Tables 2 and 3, respectively. Mannose triflate precursor, Cryptand 222 (Kryptofix 222 (K222)), and quaternary methylammonium (QMA) cartridges were purchased from ABX (Advanced Biochemical Compounds, Radeberg, Germany); 1 N hydrochloric acid (HCl) was purchased from Fisher Scientific (Pittsburg, PA, USA); and anhydrous acetonitrile (MeCN) and all other reagents were purchased from Sigma-Aldrich (Milwaukee, WI, USA). Reagents and solvents were used as received, and all water was purified to 18 MΩ and 0.1-μm filtered. Alumina-N (preconditioned with 10 mL water) and C18 (preconditioned with 5 mL ethanol and then 20 mL water) were purchased from Waters (Milford, MA, USA). Strong cation exchange (SCX) cartridge was purchased from GRACE (Deerfield, IL, USA) and was preconditioned with 10 mL of water. Ion retardation resin (AG11A8) was purchased from Bio-Rad (Hercules, CA, USA) and stored in slurry with 0.5 M sodium chloride.

**Table 2:** **Reagents for the synthesis of [**^**18**^**F]FDG**

**Position**^**a**^	**Name**	**Description**
2	Eluent	1 mg K_2_CO_3_ in 0.3 mL water; 10 mg K222 in 0.5 mL acetonitrile
3	MeCN-1	1.0 mL acetonitrile
5	MeCN-2	1.0 mL acetonitrile
6	Mannose triflate	30 mg mannose triflate dissolved in 1.0 mL acetonitrile
7	HCl	1.0 mL of 1 N HCl
8	Water-1	2.5 mL water
9	Water-2	2.5 mL water

^a^All positions are for the cassette in reactor 1.

**Table 3:** **Unit operations for the synthesis of [**^**18**^**F]FDG**

**Unit operation**		**Description**
1	INITIALIZE	Initialize hardware
2	TRAPF18	Trap [^18^F]fluoride for 120 s using 3 psi inert gas
3	ELUTEF18	Flow eluent through elute path for 120 s using 3 psi inert gas
4	EVAPORATE	Evaporate reactor 1 at 110°C for 300 s using 15 psi inert gas
5	ELUTEF18	Flow MeCN-1 through elute path for 90 s using 3 psi inert gas
6	EVAPORATE	Evaporate reactor 1 at 110°C for 120 s using 10 psi inert gas
7	ADD	Add MeCN-2 to reactor 1
8	EVAPORATE	Evaporate reactor 1 at 110°C for 120 s using 10 psi inert gas and cool to 30°C
9	ADD	Add mannose triflate to reactor 1
10	REACT	React reactor 1 at 130°C for 300 s and cool to 35°C with an additional 120s cooling delay
11	EVAPORATE	Evaporate reactor 1 at 110°C for 120 s using 10 psi inert gas
12	ADD	Add HCl to reactor 1
13	REACT	React reactor 1 at 130°C for 300 s and cool to 35°C with an additional 120-s cooling delay
14	TRANSFER	Transfer the contents of reactor 1 through the purification cartridge to the collection vial. Push with 10 psi inert gas for 30 s
15	ADD	Add Water-1 to reactor 1
16	TRANSFER	Transfer the contents of reactor 1 through the purification cartridge to the collection vial. Push with 10 psi inert gas for 30 s
17	ADD	Add Water-2 to reactor 1
18	TRANSFER	Transfer the contents of reactor 1 through the purification cartridge to the collection vial. Push with 10 psi inert gas for 45 s

**Figure 7:**
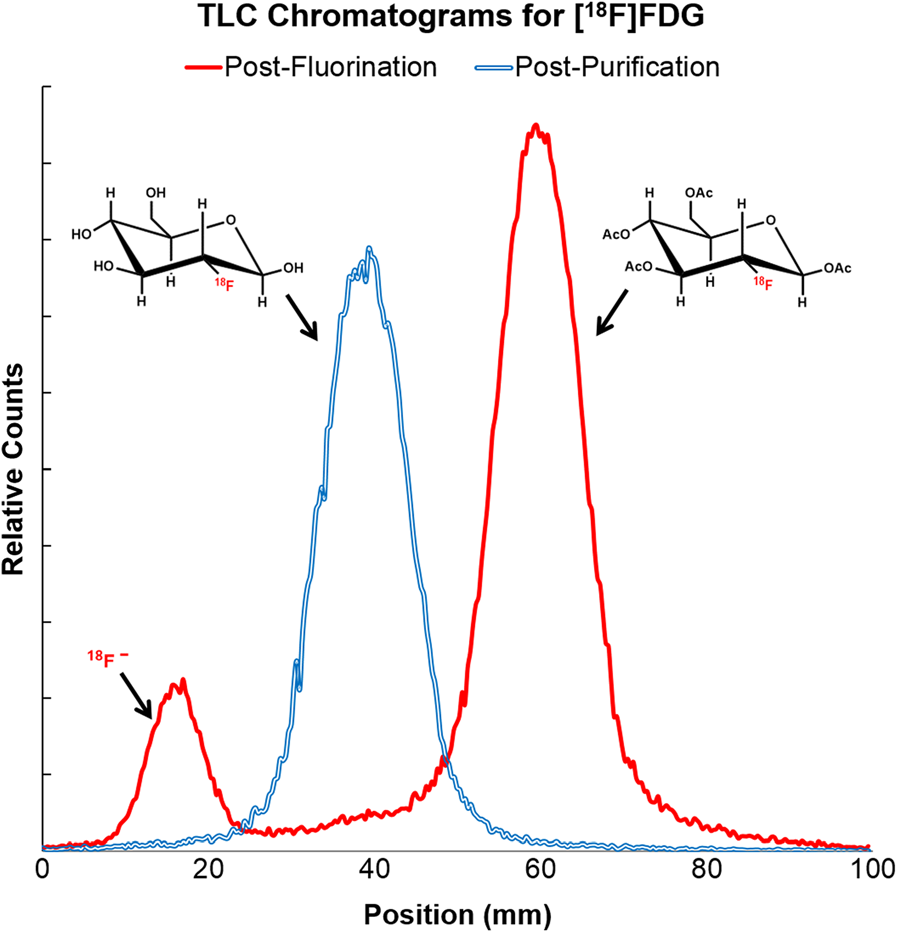
**TLC chromatograms collected post-fluorination and post-purification during an example synthesis of [**^**18**^**F]FDG.**

After synthesizer initialization, [^18^F]fluoride produced on an RDS-112 cyclotron (Siemens, Munich, Germany) was trapped on a QMA cartridge and then eluted into reaction vessel 1 (unit operations 1 to 3, Table 3). The [^18^F]fluoride solution was then dried, and two azeotropic drying steps were performed with acetonitrile (unit operations 4 to 8). A 30-mg mannose triflate precursor dissolved in 1 mL MeCN was then added, and the solution reacted at 130°C for 5 min (unit operations 9 to 10). To demonstrate the ability to take intermediate samples, the ‘INSTALL’ unit operation was placed after the fluorination reaction (after unit operation 10, not listed), and a sample was taken for thin-layer chromatography (TLC) analysis using 95% MeCN in water (*v*/*v*) (Figure 7). The solution was subsequently dried at 110°C to remove the MeCN (unit operation 11). After deprotection with HCl (unit operations 12 to 13), the crude product was purified (SCX, ion retardation, Alumina-N, and C18) using the TRANSFER unit operation (unit operation 14) and subsequently rinsed (twice) by adding water to the reaction vessel and transferring it through the purification cartridges (unit operations 15 to 18) and a sterile 0.22-μm filter into a sterile vial. A sample was taken for standard quality assurance testing at our clinical facility.

The details of reagents and synthesis program for 2-deoxy-2-[^18^F]fluoro-β-d-arabinofuranosyl (d-[^18^F]FAC) and 2-deoxy-2-[^18^F]flouro-5-methyl-1-β-l-arabinofuranosyl uracil (l-[^18^F]FMAU) are reported elsewhere [29]. The synthesis programs and results for additional probes are currently being compiled and will be published separately.

### Software evaluation

To evaluate the ease of programming synthesis protocols, programs were created for several known probes (Table 4), and the length of the programs were compared to programs supplied by the manufacturer on several commercially available radiosynthesizers from different vendors.

**Table 4:** Number of operations required to synthesize common probes

**System**	**Probe**	**Operation count**
ELIXYS	[^18^F]FDG	18 unit operations
ELIXYS	d-[^18^F]FAC	42 unit operations
ELIXYS	l-[^18^F]FMAU	42 unit operations
ELIXYS	[^18^F]SFB	15 unit operations
ELIXYS	[^18^F]FLT	15 unit operations
ELIXYS	[^18^F]Fallypride	12 unit operations
Synthera	[^18^F]FDG	227 program steps
Synthera	[^18^F]FLT^a^	241 program steps
Synthera	[^18^F]SFB^a^	206 program steps
FASTlab	[^18^F]FDG	335 program steps
Explora RN	[^18^F]FLT	72 program steps^b^

[^18^F]FDG, 2-[^18^F]fluoro-2-deoxy-d-glucose; d-[^18^F]FAC, 2-deoxy-2-[^18^F]fluoro-β-d-arabinofuranosyl; l-[^18^F]FMAU, 2-deoxy-2-[^18^F]flouro-5-methyl-1-β-l-arabinofuranosyl uracil; [^18^F]SFB, *N*-succinimidyl-4-[^18^F]fluorobenzoate; [^18^F]FLT, [^18^F]fluorothymidine; [^18^F]Fallypride, (*S*)-*N*-((1-allyl-2-pyrrrolidinyl)methyl)-5-(3-[^18^F]fluoropropyl)-2,3-dimethoxybenzamide. ^a^The [^18^F]FLT and [^18^F]SFB synthesis protocols for IBA Synthera® were developed at UCLA; ^b^Number of program steps before HPLC purification.

User-friendliness was assessed using a small sample group of six students having no prior experience with automated radiosynthesizers. The students were given a brief introduction to the synthesizer during a class lecture and were split into groups for a working session in the radiochemistry lab. Each group was provided a description of a simple chemical synthesis comparable to what is commonly found in the literature, along with a list of the available unit operations similar to Table 1. The proposed synthesis was first written out by hand, then programmed into the ELIXYS using the client interface, and finally run on the actual instrument with minimal assistance. For reasons of safety, no radioactivity was used in the syntheses.

## Results and discussion

Using the client programming interface, we developed a program for the synthesis of [^18^F]FDG, the most widely used PET probe, that requires a total of 7 reagents (Table 2) and 18 unit operations (Table 3). The concept of unit operations abstracts the steps of the synthesis from the details of the hardware to such an extent that almost no information about the engineering design of the radiochemical synthesizer is needed to understand the synthesis protocol; indeed, very few details about the underlying hardware can be gleaned from the program alone. The small number of unit operations required to synthesize [^18^F]FDG on the ELIXYS hides the complexity of the underlying system from the user; these 18 unit operations translate into 476 low-level hardware operations (e.g., individual motions of reaction vessels and switching of valves). For comparison, similar synthesis protocols for [^18^F]FDG on IBA's Synthera® and GE's FASTlab, synthesizers that were programmed with the traditional paradigm of scripting low-level hardware operations, require a total of 227 and 335 program steps to be defined by the user, respectively (Table 4). It is important to note that there are small differences between the ELIXYS and Synthera® [^18^F]FDG synthesis protocols (e.g., number of azeotropic drying steps, acid vs. base hydrolysis, cartridges for purification) and that the ELIXYS protocol has not been optimized. [^18^F]FDG produced on ELIXYS passed all quality assurance tests (e.g., radiochemical purity, pH, Kryptofix 222, pyrogenicity, sterility, residual solvent levels, TLC retardation factor (*R*_f_) comparison with cold standard, and radioisotope identity). Decay-corrected radiochemical yield of 65% ± 2% (*n* = 3) was obtained for this 45-min synthesis. The uncorrected yield of 52% is slightly lower than the 60% reported in IBA's product literature [34], but optimization could improve the final yield.

It was found that the same set of unit operations was sufficient to synthesize several simple probes in 12 to 15 steps including *N*-succinimidyl-4-[^18^F]fluorobenzoate ([^18^F]SFB), [^18^F]fluorothymidine ([^18^F]FLT), and (*S*)-*N*-((1-allyl-2-pyrrrolidinyl)methyl)-5-(3-[^18^F]fluoropropyl)-2,3-dimethoxybenzamide ([18F]Fallypride). The performance of these syntheses was comparable to literature reports and will be published separately. Consistent with the results for [^18^F]FDG, the ELIXYS program for [^18^F]SFB consisted of only 15 unit operations compared to the 206-step program to produce [^18^F]SFB with Synthera®. Similarly, synthesis programs for [^18^F]FLT on ELIXYS and Siemen's Explora RN required 15 and 87 steps, respectively. Complex probes that utilize all three reaction vessels, such as d-[^18^F]FAC and l-[^18^F]FMAU required more steps than one-pot syntheses (i.e., 42 steps). d-[^18^F]FAC and l-[^18^F]FMAU were produced with acceptable quality and with yield and synthesis time comparable to literature reports [29]. Since no other commercial system is capable of synthesizing these probes with comparable yields, program lengths cannot be compared to other systems but would likely be substantially shorter. Of particular importance is the observation that no tweaking beyond the adjustment of parameters in the high-level unit operations was required to produce the variety of probes above with yields comparable to those obtained on more traditional radiosynthesizers. A manuscript detailing several different chemical syntheses on the ELIXYS system will be published separately.

The consistent finding that program length is considerably shorter on the ELIXYS system suggests that it will be easier and faster to create and edit synthesis programs compared to conventional software approaches. It is also likely that these programs will have fewer bugs and be easier to debug.

As another measure of the ease of programming, six students with no prior experience with automated radiosynthesizers took part in an exercise to estimate the learning curve of the software. The students were able to complete programming the system within 1 h and subsequently set up and execute the program on the ELIXYS synthesizer with very little assistance. While not a controlled trial, this exercise was informative both because the students found the system easy to use overall and because it highlighted several areas of the user interface that were less intuitive than originally thought. The observations from this exercise will be used to enhance the client software.

When running a synthesis program, the ability to shorten or extend the duration of timed unit operations such as reaction or evaporation steps was found to be advantageous for synthesis development and optimization. In combination with the live video from the active reactor, we found this feature useful in dealing with situations such as determining the optimum length of time to evaporate to a specific level, measuring the time required to reach the endpoint of a reaction where a visible change (e.g., color) is associated with reaction completion, determining the required time to transfer a new type or volume of liquid from a reagent vial to the reaction vessel, and measuring the length of time required for the contents of a reaction vessel to be purified through a new type of cartridge. The actual duration of each adjustable step is recorded to the synthesis run history to enable the user to review how long each step actually took as well as to replay a previous program run.

In terms of reliability, in over 100 production runs, we have not observed any failures due to software error. Furthermore, as designed, we have noted that the production run has continued without interruption in several cases where a client device failed, e.g., due to running out of battery power, or when network connectivity was temporarily disrupted.

Although ELIXYS is currently supporting only preclinical PET probe production, we are in the process of implementing several software features to enable compliance with cGMP guidelines and plan to place the upgraded system into a laboratory that supports clinical production of PET probes under both 21 CFR 212 and USP 823. Additionally, Sofie Biosciences is working on amending the CMC section of an ongoing phase 1 clinical trial for d-[^18^F]FAC to include ELIXYS for the production of this probe.

## Conclusions

We have developed a software package that operates the ELIXYS automated radiosynthesizer and is designed to shift the programming paradigm away from the approach of specifying low-level hardware operations that requires a detailed understanding of the underlying system. The programming model presented here allows radiochemists to string together a sequence of high-level unit operations that are designed to make intuitive sense without requiring any more knowledge of the underlying hardware than necessary. We have found the current set of unit operations to be sufficient to enable the synthesis of a variety of PET probes of varying complexity without needing to resort to low-level hardware operations, simplifying the task of programming and significantly reducing the length of synthesis programs compared to other synthesizer software.

Additionally, we found that declaring the reagents at the beginning of synthesis development and thereafter referring to them by name is more convenient than having to remember the actual location of each installed reagent. We have also found that the tools for developing new synthesis protocols, such as the ability to view a live video feed of the reaction vessel and to alter the length of timed steps during the synthesis run, were effective in dealing with portions of new synthesis protocols where the optimal duration was not known *a priori*.

## Competing interests

RMvD and MM own equity in Sofie Biosciences, and RMvD is a consultant for Sofie Biosciences. SBC and KMQ have worked for Sofie Biosciences as paid consultants. ML declares that he has no competing interests.

## Authors’ contributions

All authors - SBC, KMQ, ML, MDM, and RMvD - were involved in the design of the software architecture and user interface. KMQ performed low-level programming of the PLC; SBC and KMQ programmed the core server applications, and SBC programmed the client application. All authors contributed to the testing and refinement of the complete software package. ML developed and performed the [^18^F]FDG synthesis. SBC, ML, and RMvD wrote the manuscript. All authors read and approved the final manuscript.

## Supplementary Material

Additional file 1 Detailed list of low-level hardware operations that make up each high-level unit operation. Click here for file
