# Supplementary material for: Simplified programming and control of automated radiosynthesizers through unit operations
Source: EJNMMI Res. 2013 Jul 15;3:53. doi: 10.1186/2191-219X-3-53 (PMC3717018; doi:10.1186/2191-219X-3-53)
Supplement: Additional file 1 — Detailed list of low-level hardware operations that make up each high-level unit operation. [file 2191-219X-3-53-S1.doc]

**Supplemental data**

**Add unit operation.** Adds a reagent to a reaction vessel.

Parameters:

- reactor – Reactor where the reagent will be added.
- reagent – Name of the reagent to add.
- delivery position – Choice of two delivery positions.
- delivery time – Time to add reagent in seconds.
- delivery pressure – Pressure to apply to reagent in PSI.

Low level operation:

# Adjust pressure

Set regulator 1 to delivery pressure

# Move reactor to add position

Lower reactor

Enable reactor robot

Move reactor robot to add position

Raise reactor

Disable reactor robot

# Move reagent vial to delivery position

Open gripper

Raise gripper

Raise gas transfer

Enable reagent robot

Move reagent robot to reagent position

Lower gripper

Close gripper

Raise gripper

Move reagent robot to delivery position

Lower gas transfer

Open gas transfer valve

Lower gripper

# Deliver reagent

Wait for delivery time to elapse

# Return vial to reagent position

Raise gripper

Close gas transfer valve

Raise gas transfer

Move reagent robot to reagent position

Lower gripper

Open gripper

Raise gripper

Move reagent robot to home

**Evaporate unit operation.** Evaporates the contents of a reaction vessel.

Parameters:

- reactor – Reactor that will be heated while gas and vacuum are applied.
- evaporation time – Time to evaporate in seconds.
- evaporation temperature – Evaporation temperature in Celsius.
- evaporation pressure – Pressure to apply in PSI.
- final temperature – Final reactor temperature after evaporation in Celsius.
- stir speed – Speed of the stir motor.
- stop at temperature – Flag that indicates if the stir motor will stop once the reactor reaches the evaporation temperature.

Low level operation:

# Adjust pressure

Set regulator 1 to 30% of evaporation pressure

# Move reactor to evaporation position

Lower reactor

Enable reactor robot

Move reactor to evaporate position

Raise reactor

Disable reactor robot

# Start stirring

Set reactor motor to stir speed

# Move robot to supply gas and vacuum

Raise gripper

Raise gas transfer

Enable reagent robots

Move reagent robot to evaporate position

Lower gas transfer

# Heat reactor

Open gas transfer valve

Start vacuum system

Set reactor heater target to evaporation temperature

Turn on reactor heaters

Wait to reach evaporation temperature

If stop at temperature:

Set reactor stir speed to zero

# Evaporate, ramping the pressure during the first half

Ramp regulator 1 to evaporation pressure over 50% of evaporation time

Wait for the remaining evaporation time to elapse

# Cool reactor

Turn off reactor heaters

Start cooling system

Wait to reach final temperature

Stop cooling system

If not stop at temperature:

Set reactor stir speed to zero

# Complete

Lower reactor

Close gas transfer valve

Stop vacuum system

Raise gas transfer

Move reagent robot to home

**Transfer unit operation.** Transfers the contents of one reactor to either a waste vial (trap mode) or another reactor (elute mode). A cartridge can optionally be installed for purification.

Parameters:

- source reactor – Reactor that contains the solution to be transferred.
- target reactor – Reactor that is plumbed to receive the solution from the source reactor.
- transfer mode – Mode that indicates if the solution should be sent to waste or the target reactor.
- transfer pressure – Pressure to apply when transferring in PSI.
- transfer time – Time to transfer in seconds.

Low level operation:

# Move source reactor to transfer position while stirring

Set source reactor stir speed to 500

Lower source reactor

Enable source reactor robot

Move source reactor to transfer position

Raise source reactor

Disable source reactor robot

Set source reactor stir speed to zero

# Move target reactor to add position

Lower target reactor

Enable target reactor robot

Move target reactor to add position

Raise target reactor

Disable target reactor robot

# Move robot to supply gas

Raise gripper

Raise gas transfer

Enable reagent robots

Move reagent robot to transfer position

Lower gas transfer

# Transfer

If transfer mode is trap:

Set transfer stopcock for reactor to trap position

Else transfer mode is elute:

Set transfer stopcock for reactor to elute position

Set regulator 1 to transfer pressure

Open gas transfer valve

Wait for transfer time to elapse

Close gas transfer valve

Set transfer stopcock for reactor to default position

# Home robot

Raise gas transfer

Move reagent robot to home

**React unit operation.** Seals the reactor and heats to facilitate a chemical reaction.

Parameters:

- reactor – Reactor to be heated.
- reaction position – Choice of two distinct reaction stations to prevent cross-contamination.
- reaction time – Time to heat in seconds.
- reaction temperature – Temperature of reaction in Celsius.
- final temperature – Final reactor temperature after reaction in Celsius.
- cooling delay – Additional time to cool after reactor reaches final temperature.
- stir speed – Speed of the stir motor.
- stop at temperature – Flag that indicates if the stir motor will stop once the reactor reaches the reaction temperature.

Low level operation:

# Move reactor to react position

Lower reactor

Enable reactor robot

Move reactor to react position

Raise reactor

Disable reactor robot

# Start stirring

Set reactor motor to stir speed

# Heat reactor

Set reactor heater target to reaction temperature

Turn on reactor heaters

Wait to reach reaction temperature

If stop at temperature:

Set reactor stir speed to zero

# React

Wait for reaction time to elapse

# Cool reactor

Turn off reactor heaters

Start cooling system

Wait to reach final temperature

Wait until cooling delay has elapsed

Stop cooling system

If not stop at temperature:

Set reactor stir speed to zero

**Prompt unit operation.** Pauses the sequence run and prompt the user.

Parameters:

- message – Message to display to the user.

Low level operation:

# Wait for user to acknowledge message

Wait for user input

**Initialize unit operation.** Initializes the system hardware.

Low level operation:

# Initialize valves

Close gas transfer valve

For reactors 1, 2 and 3:

Lower reactor

Set transfer stopcock for reactor to default position

Set F18 stopcocks for reactor to default positions

Close F18 loading valve

# Initialize cooling, vacuum, heaters and stir motors

Stop cooling system

Stop vacuum system

For reactors 1, 2 and 3:

Turn off reactor heaters

Set reactor stir speed to zero

# Set pressures

Set regulator 1 to default gas transfer pressure

Set regulator 2 to default pneumatic pressure

# Raise and open gripper

Raise gripper

Open gripper

Raise gas transfer

# Home the robots

Home reagent robots

For reactors 1, 2 and 3:

Home reactor robot

# Lock the robots into the install positions

For reactors 1, 2 and 3:

Lower reactor

Enable reactor robot

Move reactor to install position

Raise reactor

Disable reactor robot

**Install unit operation.** Moves the reactor to the install position and optionally prompts the user.

Parameters:

- reactor – Reactor to move to the install position.
- message – Message to display to the user.

Low level operation:

# Move reactor to the install position

Lower reactor

Enable reactor robot

Move reactor to install position

Raise reactor

Disable reactor robot

# Wait for user to acknowledge message

If message:

Wait for user input

**Comment unit operation.** User comment in a synthesis that can optionally be broadcast to subscribers.

Parameters:

- comment – User-defined synthesis comment.
- broadcast flag – Flag that indicates if the comment should be broadcast to subscribers.

Low level operation:

# Broadcast message to subscribers (sent via email or text message)

If broadcast flag:

Broadcast comment

**TrapF18 unit operation.** Traps [18F]fluoride on a QMA cartridge.

Parameters:

- reactor – Reactor where the QMA cartridge is installed.
- cyclotron flag – Flag that indicates if the Elixys or the cyclotron will load the target volume.
- trap time – Load duration in seconds (non-cyclotron mode only).
- trap pressure – Load pressure in PSI (non-cyclotron mode only).

Low level operation:

# Vent pressure

Vent pressure by setting regulator 1 to zero

# Move reactor and stopcock

Lower reactor

Enable reactor robot

Move reactor to add position

Raise reactor

Disable reactor robot

Set F18 stopcocks for reactor to trap position

If cyclotron flag:

# Wait for the user to indicate the cyclotron has delivered the target

Wait for user input

Else:

# Push the target

Open F18 loading valve

Ramp regulator 1 to trap pressure over 5 seconds

Wait for trap time to elapse

Close F18 loading valve

Set F18 stopcocks for reactor to default position

**EluteF18 unit operation.** Uses a reagent to elute the [18F]fluoride off a QMA cartridge.

Parameters:

- reactor – Reactor where the QMA cartridge is installed.
- elution time – Elution duration in seconds.
- elution pressure – Elution pressure in PSI.
- elution reagent – Reagent that will be used to elute the QMA cartridge contents.

Low level operation:

# Vent pressure

Vent pressure by setting regulator 1 to zero

# Move reactor

Lower reactor

Enable reactor robot

Move reactor to add position

Raise reactor

Disable reactor robot

Set F18 stopcocks for reactor to elute position

# Pick up elution reagent

Open gripper

Raise gripper

Raise gas transfer

Enable reagent robots

Move reagent robot to elution reagent position

Lower gripper

Close gripper

Raise gripper

Move reagent robot to elute position

Lower gas transfer

Open gas transfer valve

Lower gripper

# Elute

Ramp regulator 1 to elution pressure over 5 seconds

Wait for elution time to elapse

# Return elution reagent

Raise gripper

Close gas transfer valve

Raise gas transfer

Move reagent robot to elution reagent position

Lower gripper

Open gripper

Raise gripper

Move reagent robot to home

Set F18 stopcocks for reactor to default position

**Mix unit operation.** Mixes the contents of a reactor by stirring.

Parameters:

- reactor – Reactor to be mixed.
- mix time – Mixing duration in seconds.
- stir speed – Speed of stir motor.

Low level operation:

# Stir

Set reactor motor to stir speed

Wait for mix time to elapse

Set reactor stir speed to zero

**Move unit operation.** Moves a reactor to the desired position.

Parameters:

- reactor – Reactor to be moved.
- position – Target reactor position.

Low level operation:

# Move reactor

Lower reactor

Enable reactor robot

Move reactor to position

Raise reactor

Disable reactor robot

**ExternalAdd unit operation.** Allows the user to externally add a reagent via tubing.

Parameters:

- reactor – Reactor where the reagent will be added externally.
- reagent name – Name of the reagent that will be displayed to the user.

Low level operation:

# Move reactor to add position

Lower reactor

Enable reactor robot

Move reactor to add position

Raise reactor

Disable reactor robot

# Wait for user to indicate that the external reagent has been added

Wait for user input
